# Going beyond ejection fraction - CMR assessment of ventricular-vascular coupling and LV remodeling predicts diastolic dysfunction in advanced ischemic cardiomyopathy

**DOI:** 10.1186/1532-429X-15-S1-P193

**Published:** 2013-01-30

**Authors:** Joao L Cavalcante, Zoran B Popovic, Rory Hachamovitch, Milind Y Desai, Scott D Flamm, Thomas Marwick, Deborah Kwon

**Affiliations:** 1 Heart and Vascular Institute, University of Pittsburgh, Pittsburgh, PA, USA; 2 Heart and Vascular Institute, Cleveland Clinic Foundation, Cleveland, OH, USA; 3 Imaging Institute, Cleveland Clinic Foundation, Cleveland, OH, USA; 4 Menzies Research Institute, University of Tasmania, Hobart, TAS, Australia

## Background

Increased ventricular and arterial stiffness is associated with diastolic dysfunction (DDFx) in patients with heart failure and preserved systolic function. Limited information is available regarding the impact of aortic biomechanics and the ventricular-vascular coupling (VVC) on DDFx in those patients with advanced ischemic cardiomyopathy (ICM). In addition, it is not known if cardiac magnetic resonance (CMR) measurements of LV remodeling (sphericity and scar burden) can also contribute to prediction of DDFx in these patients. We sought to examine the relationship between aortic biomechanical properties (ascending and descending distensibility, arch pulse wave velocity), ventricular-ventricular coupling (defined as the ratio between LV end-systolic elastance and effective arterial elastance), LV remodeling assessed by CMR and diastolic function assessed by echocardiography in patients with advanced ICM.

## Methods

Patients were selected if they had undergone TTE and CMR studies within 7 days (median=1 day). 354 patients with LVEF ≤ 40% and ≥ 70% stenosis in ≥1 coronary artery but without prior mitral valve surgery, fused E/A waves, atrial fibrillation or > moderate mitral regurgitation were screened. Of those, 84 patients were excluded due to poor CMR image quality from artifacts and/or suboptimal temporal resolution. A total 270 charts were reviewed for demographic and laboratorial data. Diastolic function assessment was performed as per guidelines. Aortic biomechanics were measured using previously validated software (ARTFUN, INSERM U678, Paris, France) using semi-automated tracing of aortic contours with phase-contrast images and through-plane velocity encoding of the ascending and descending aorta. CMR evaluation also included long and short axis assessment of LV sphericity and function respectively on balanced steady state free precession images along with assessment of myocardial scar (on phase-sensitive inversion recovery DHE-CMR sequence ~ 10-20 minutes). Multivariate linear regression analysis was done to identify the independent predictors of DDFx.

## Results

Males represented 76% of the cohort with a mean age of 62 ± 10 years. Mean LVEF was 23 ± 5% and DDFx was classified as either: stage 1 (44%), stage 2 (25%) or stage 3 (31%). The independent predictors of impaired diastolic function (stage > 1) are listed on Table 1.

**Table 1:** Multivariate predictors of LV diastolic dysfunction (*)

		Unstandardized Coefficients	Standardized Coefficients		95.0% Confidence Interval for B	
Linear Regression Model	B	Std Error	Beta	P value	Lower Bound	Upper Bound

WC	-.524	.156	-.196	0.001	-.831	-.217
Diabetes Mellitus	.346	.094	.247	<0.0001	.160	.531
Vena contracta	.877	.270	.185	0.001	.345	1.409
Age	-0.12	.004	-.151	.007	-.021	-.003
Gender male	-.231	.108	-.117	.033	-.445	-.018
LV Sphericity	1.322	.589	.129	.025	.163	2.481
Scar Burden (#)	.006	.003	.111	.045	.000	.011

(*) After adjusting for age, hypertension, dyslipidemia, QRS duration, ascending and descending aorta distensibility, arch PWV, end-systolic volume index, body surface area, LV mss index, coronary artery disese severity, scar burden. VVC = ventricular-vascular coupling. (#) Scar burden was assessed using the 17-segment left ventricular model.

## Conclusions

In patients with advanced ICM, CMR assessment of VVC, LV sphericity and scar burden are independent predictors of DDFx. Aortic biomechanical properties are not independently associated with diastolic dysfunction.

## Funding

None.

